# Do We Belittle Essential Tremor by Calling It a Syndrome Rather Than a Disease? Yes

**DOI:** 10.3389/fneur.2020.522687

**Published:** 2020-10-15

**Authors:** Abhishek Lenka, Elan D. Louis

**Affiliations:** ^1^Department of Neurology, MedStar Georgetown University Hospital, Washington, DC, United States; ^2^Department of Neurology, University of Texas Southwestern, Dallas, TX, United States

**Keywords:** essential tremor (ET), disease, syndrome, tremor, movement disorder

## Abstract

Essential tremor (ET) is among the most prevalent neurological diseases. Appreciation in recent years of a richer tremor phenomenology, additional motor and non-motor features, variability in the natural course of tremor, associations with a host of other neurological conditions, and etiological and pathophysiological heterogeneity have resulted in general awareness of the clinical richness of ET. Along with this evolving view of ET have surfaced several conundrums regarding nomenclature. One of these is whether ET should be labeled a “syndrome” or “disease.” Here, we revisit the classical definitions of “syndrome” and “disease” and discuss ET in this context. Considering the characteristics of “disease” and “syndrome” and evaluating the characteristics of ET, it seems to fit more into the “disease” construct. There are several reasons: There is considerable knowledge of the underlying etiologies and pathophysiology of ET, in numerous studies ET has been linked with other neurological conditions, the condition is progressive and deteriorative, and therapeutic approaches are grounded in an understanding of disease mechanisms and its associated neuroanatomy. Moreover, the etiological–pathological–clinical heterogeneity suggests that ET should be regarded as a “family of diseases” more appropriately termed “the essential tremors.” This nomenclatural issue is not a mere matter of words; public health implications are numerous. A condition with the label “syndrome” may not be recognized as a serious problem, may be plagued by diminished public awareness, and may not garner funds for research that a condition with the label “disease” or “diseases” would. ET should be regarded as a family of diseases.

## Introduction

Essential tremor (ET) is one of the common neurological diseases. Our knowledge of its clinical phenomenology, natural course, and pathogenesis has expanded considerably during the past several decades (1). ET was considered a monosymptomatic illness, characterized only by tremor. Subsequent identification of a richer tremor phenomenology, additional motor features, a repertoire of non-motor features, variability in rates of progression, associations with a host of other neurological diseases, and etiological and pathophysiological heterogeneity have resulted in a greater general awareness of the clinical richness of ET (1). Along with this evolving view of ET have surfaced several conundrums regarding nomenclature (2–4). One of these is whether ET should be labeled a “syndrome” or “disease.” More specifically, although it is becoming increasingly clear to most experts that “ET” is a phenotypically heterogeneous condition or set of conditions, there is a debate as to whether to conceptualize ET as a “syndrome” or a “disease.” Some experts are of the opinion that ET is a “syndrome” (5, 6), whereas others are of the opinion that ET is a “disease” or “family of diseases” or “group of diseases.” (7–9). Interestingly, a similar conundrum may be found with respect to epilepsy (10), where the nomenclatural issues and their repercussions have been discussed in detail, and the public health implications have been well articulated—a condition with the label “disorder” or “syndrome” may not be recognized as a serious problem, may remain in the shadows, may be plagued by diminished public awareness, and may not garner funds for research that a condition with the label “disease” would (10).

In this review, we revisit the classical definitions of these terms, “syndrome” and “disease,” and discuss ET in this context.

## Definitions of “Syndrome” and “Disease”

### Syndrome

Although several definitions have been put forth for “syndrome” and “disease,” there are no universally accepted definitions and no formally derived inclusion or exclusion criteria. Moreover, definitions have changed over time (11). This makes the current debate challenging.

The term “syndrome” is derived from Greek (“*syn”* together and “*dromus”* a course), and it means “a running together or concurrence.” A syndrome is a recognizable complex of symptoms and physical findings that indicate a specific condition for which a direct cause is not necessarily understood (12). In other words, syndromes describe a specific collection of symptoms and signs which recurrently co-occur. Although classically, the word “syndrome” has been applied to conditions with no immediately recognizable etiopathogenesis (e.g., Angelman syndrome, West syndrome), there are conditions that have been labeled “syndromes” despite considerable development in our understanding of their pathogenesis (e.g., Guillain-Barré syndrome). However, in general, once medical science identifies the causative agents (i.e., etiology) and pathogenesis of a particular condition, the term “syndrome” tends to be replaced by “disease” (12–17). For example, with advances in knowledge, mucocutaneous lymph node syndrome (Kawasaki syndrome) is no longer viewed as a syndrome but as a proper disease (Kawasaki disease) (12).

### Disease

Occasional views have been put forth that the concept “disease” is unnecessary for clinical thinking or clinical decision making (18); however, such views are not mainstream, and for the most part, the value of the concept “disease” is indisputable to patients, healthcare providers, and society. “Disease” is a fluid concept influenced by sociocultural attitudes and political motivations, which are prone to change with time and in response to new medical and scientific discoveries (19). What counts or does not count as a disease fluctuates over time, partly as a result of increasing expectations of health, partly due to changes in diagnostic ability, and also for social and economic reasons (20). For example, osteoporosis, which was considered a symptom/sign of aging, was, by the mid-1990s, regarded as a disease. This nomenclatural issue has consequences for sufferers' sense of whether they are “normally old” or “ill” and, more concretely, for their ability to have treatment reimbursed by health service providers (20). The other example is homosexuality, which was once regarded as a disease secondary to endocrine abnormalities or as an organic mental disorder. It was officially “de-pathologized” by the American Psychiatric Association, in 1974, as homosexual behavior is a widely prevalent aspect of human sexuality and not a pathologic entity (20, 21).

**Figure 1:**
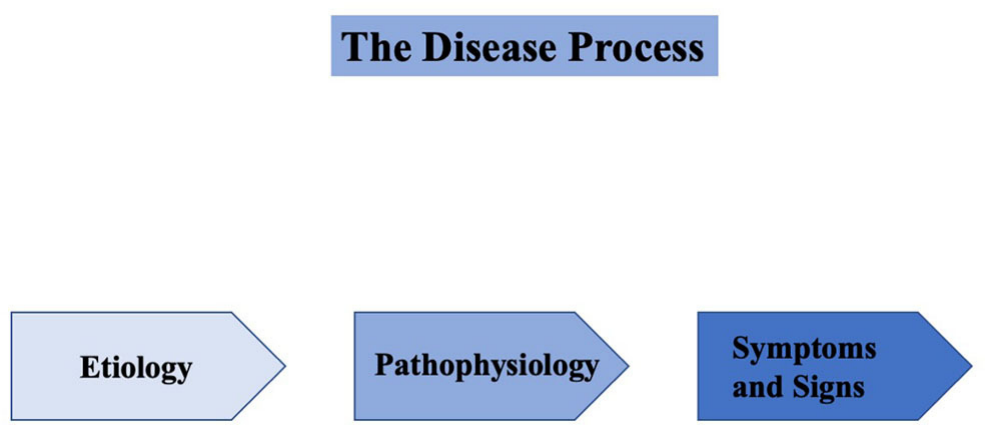
Diseases are entities that have identifiable causes (etiologies), underlying pathophysiologies (sometimes with associated organ-based changes), and associated symptoms and signs.

However, the discussion of what constitutes a disease is not merely an abstract or sociological one. Although it is clear that “syndrome” and “disease” overlap in terms of the requirement of recurring symptoms and signs, the term “syndrome” refers to recurrent co-occurrence of a set of signs and symptoms in the absence of a robust understanding of the pathogenesis and etiology, whereas the term “disease” has additional characteristics in terms of information on etiology and pathogenesis (12–17). In other words, diseases are entities that have identifiable causes and underlying pathophysiologies (sometimes with associated organ-based changes) and are more than a loose arrangement of symptoms and signs (Figure 1). Additionally, there are three other general features of disease that should be highlighted. First, disease is a state that places individuals at increased risk of adverse consequences (i.e., additional morbidity and mortality). Second, the term disease usually connotes a progressive disorder or one in which deterioration and decline occur (16). Third, based on the fact that the etiology and pathogenesis of a disease are better elucidated compared to that of a syndrome, for diseases, therapies are more often biologically based and specifically targeted toward pathogenic mechanisms.

## ET: Syndrome vs. Disease

**Figure 2:**
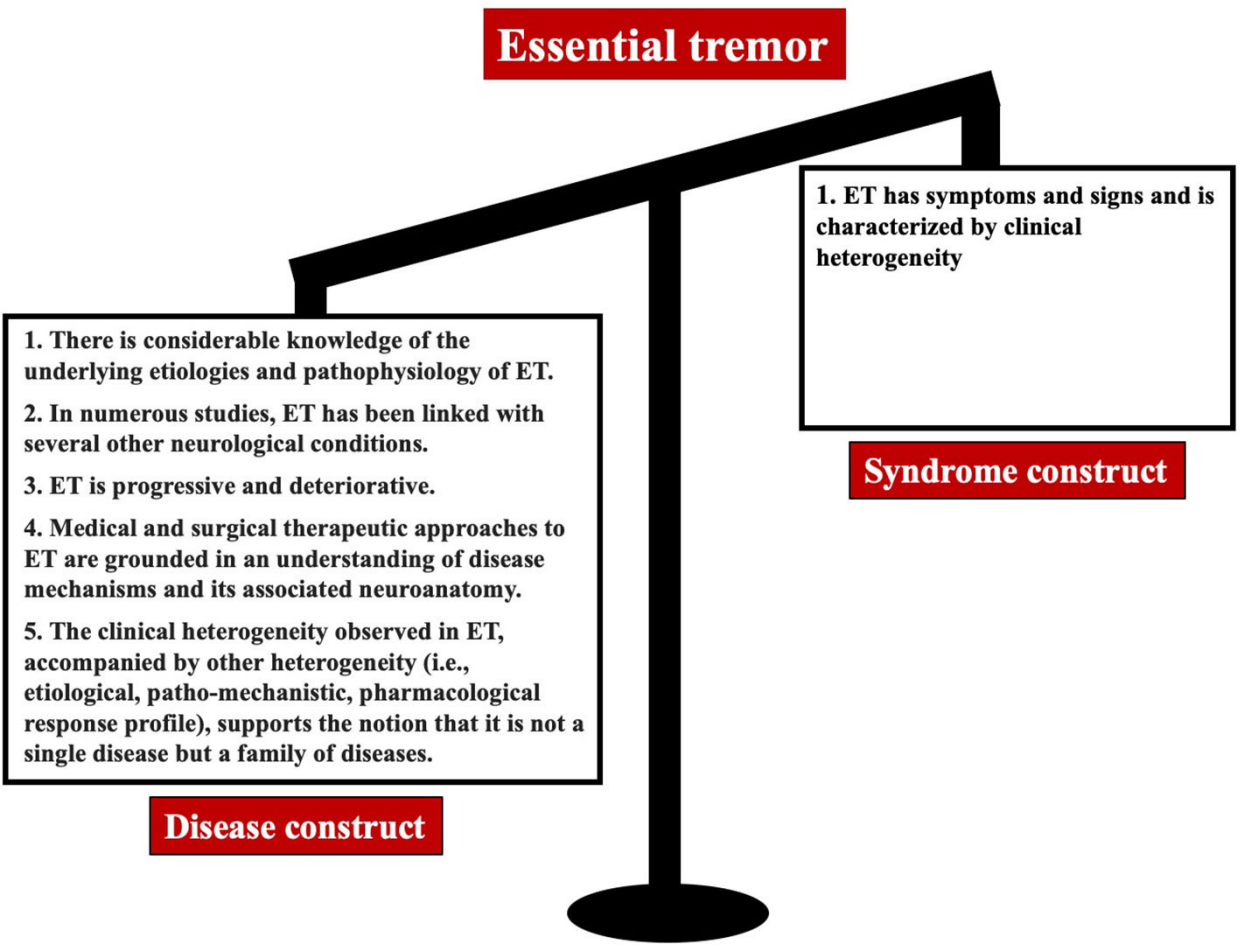
The bulk of evidence favors the view that ET is a disease or family of diseases rather than a syndrome.

Having discussed the basic concepts and definitions of “syndrome” and “disease,” we now consider whether ET better fits the “syndrome” or “disease” designation (Figure 2).

### Etiology and Pathogenesis of ET

With ET, we are dealing with more than a mere collection of symptoms and signs. This collection of symptoms and signs is linked to and grounded in etiological and pathophysiological processes, suggesting that it is a proper “disease” rather than a mere “syndrome.”

We start with a discussion of etiology. ET is not merely a collection of symptoms and signs without an apparent cause or set of causes. Both genetic and environmental factors (i.e., toxins) are identified as possible contributors to the etiology of ET. That genetic factors contribute to ET is clear. A large familial aggregation study reveals that first-degree relatives of ET patients were five times more likely to develop ET than were first-degree relatives of controls (22). Twin studies similarly demonstrate a considerable increase in disease concordance in monozygotic than dizygotic twins (22, 23). Although no major gene has been identified as of yet, several ET-linked genes are identified in ET families, and this growing collection of genes points to a clearer heterogeneity of genetic etiologies (24, 25). There is also accumulating evidence that non-genetic etiologies likely play a role in disease etiology. Several environmental toxins, which include β-carboline alkaloids (e.g., the dietary toxin harmane) and lead have been investigated, and there is growing support for the notion that these could be etiological agents (26, 27). Thus, as with Parkinson's disease (PD), in ET, both genetic and environmental factors serve as disease triggers. That is, both genetic and environmental factors are thought to launch the disease process or processes, and this, in turn, manifests clinically as symptoms and signs. Temporally, in between the prime mover (i.e., etiological factor or factors) and the symptoms and signs, is the pathophysiology (i.e., biological changes that occur once the disease process has been set in motion). A discussion of pathophysiology now follows.

Over the past several decades, advances in neuroimaging and neuropathology have provided valuable insights into the pathophysiology of ET. There is growing evidence that the underlying disease process, in all likelihood neurodegenerative, centrally involves the cerebellum. Numerous neuroimaging studies observe significant structural, functional, and metabolic alterations in the cerebellum and the cerebello–thalamo–cortical tracts in ET (28). The studies use a variety of methods, from magnetic resonance spectroscopy to volumetrics, and they suggest an underlying neuronal degeneration in the ET cerebellum (29). Postmortem studies reveal significant abnormalities in ET brains compared to matched control brains, indicating that these changes are disease-linked. The abnormalities in ET brains lie predominantly in the Purkinje cell population and include changes in the Purkinje cell dendrites (increase in dendritic swellings, pruning of dendritic architecture, loss of dendritic spines), Purkinje cell body (increase in Purkinje cell heterotopias), and Purkinje cell axon (increase in numbers of torpedoes, axonal recurrent collaterals, branching, terminal axonal sprouting, and arciform axons) (30). Changes to neighboring neuronal populations are also observed (i.e., climbing fibers and basket cells), and in properly designed studies, a reduction in the Purkinje cell population is seen (30). Along with this is the concept that there is an aberrant reduction in gamma amino butyric acid (GABA)-ergic tone in ET (31).

The above-referenced studies highlight that there are identifiable underlying causes and identifiable tissue-based processes that are disease-linked in ET. In ET, we deal with more than a mere collection of symptoms and signs. We are dealing with a collection that is linked to and grounded in specific and observable etiological and pathophysiological processes.

### Adverse Consequences of ET (i.e., Additional Morbidity and Mortality)

Disease is viewed as a state that places individuals at increased risk of adverse consequences (i.e., additional morbidity and mortality) (19). Although sometimes still debated, the overwhelming bulk of published clinical and epidemiological data support an association between ET and PD (32), and a population-based longitudinal study in Spain quantifies that patients with ET are four times more likely than controls to develop incident PD (33). Similarly, a growing number of epidemiological studies support the association between ET and mild cognitive impairment and between ET and both prevalent and incident dementia (34). Finally, although there has only been one prospective longitudinal study of mortality in ET vs. controls, that study reveals a slight but significant increased risk of mortality in ET (35). In summary, ET is a disease state that places individuals at increased risk of adverse consequences (i.e., both additional morbidities as well as increased risk of mortality).

### Progressive Disorder

“Disease” usually connotes a progressive disorder or one in which deterioration and decline occur; this is certainly the case in ET, which is slowly yet relentlessly progressive in all cases (36).

### Biologically Based Therapeutics of ET

As pathogenesis of a disease is better elucidated compared to that of a syndrome, therapies for diseases are more often biologically based and specifically targeted toward pathogenic mechanisms. The treatments for ET are increasingly biologically based and specifically targeted toward pathogenic mechanisms and/or neuroanatomic pathways. Thus, along with the older agent primidone, many of the more recently considered medications are based on the notion that GABA-ergic tone is reduced in ET, possibly as a result of changes in the Purkinje cell population although other specific molecular mechanisms are also implicated (37) and are the basis for pharmacotherapeutics. Newer generation agents, currently in testing, are similarly based on clear underlying biological considerations. Furthermore, deep brain stimulation surgery of the ventral intermediate nucleus of the thalamus and magnetic resonance image–guided focused ultrasound of the thalamus are based on the understanding that the disease is grounded in a specific neuroanatomical neuronal loop. In summary, for ET and other diseases, therapies are often biologically based and specifically targeted toward pathogenic mechanisms.

## ET: “Disease” or “Family of Diseases''?

Having reviewed the data above and highlighted the abundant support for the notion that ET is a “disease” rather than a “syndrome,” we must go one step further to discuss whether ET is a single disease or a family of diseases. In doing so, we revisit the marked heterogeneity of ET: etiological, pathological, and clinical. The etiological heterogeneity is apparent from the fact that different genes and, in some cases, no genes are associated with the emergence of ET. The heterogeneity from the pathological standpoint stems from the observation that, in contrast to changes in the Purkinje cells in the cerebellum, some ET brains were found to have an abundance of Lewy bodies that is above and beyond that normally seen in control brains (38), and some others have had neuronal inclusions (39). There is considerable evidence to highlight heterogeneity in the clinical features of ET. Heterogeneity is reported in the age at onset of tremor (e.g., bimodal pattern), distribution of tremor [e.g., higher prevalence of head tremor in female patients; (40)], presence of a family history of tremor, and response to treatment (9). Based on the presence of such multidimensional heterogeneity, involving etiology, pathogenesis, clinical features, and pharmacological response profile, it seems probable that ET is a “family of diseases” and that the term “the essential tremors” is now the appropriate one (9). It is important to note that a “family of diseases” is not the same as a “syndrome” as a family of diseases is comprised of individual diseases that each are characterized by each of the features of disease that we outline in this paper whereas a syndrome has the features, dissimilar to disease, that we also outline in this paper.

### Why Might ET Be Referred to as a “Syndrome''?

Although we point out considerable evidence in favor of ET as a “disease” construct, several experts voice opinions that it is a “syndrome” (5, 6). It is the clinical heterogeneity of ET that underlies this view. However, such heterogeneity could easily be explained by a number of factors. First, the clinical features evolve with time as patients move through different disease stages; hence, different snapshots of the disease are apparent over time (41). Second, the observed clinical heterogeneity likely is a marker that one is not dealing with only one disease but that one is dealing with a family of diseases (i.e., a constellation of clinically similar etiological–pathological–clinical entities). The members of this family likely differ with respect to environmental and genetic determinants, pathophysiological, and tissue-based changes, responses to therapies, and clinical profiles.

## Conclusion

Considering the characteristics of both “disease” and “syndrome,” it seems that ET fits more into the “disease” construct than the “syndrome” construct. We review the features of diseases, and ET fulfills each. Thus, there is considerable knowledge of the etiologies and pathogenesis of ET; in numerous studies, ET is linked with additional morbidities and/or mortality; ET is progressive and deteriorative; and therapeutic approaches, both medical and surgical, are grounded in an understanding of disease mechanisms and anatomy. Moreover, the etiological–pathological–clinical heterogeneity of ET suggests that ET should be regarded as a “family of diseases” better termed “the essential tremors.” As noted above, the issue is not merely nomenclatural; public health implications are numerous. There is no doubt that conflicts and controversies regarding the nomenclature will persist as long as we do not have absolutely clear definitions of “syndrome” and “disease.” However, considering the significant negative psychosocial and financial repercussions of ET, it seems the label “family of diseases” is apt for the time being.

## Author Contributions

AL designed and conceptualization of the work and prepared the first draft of the manuscript. EL designed and conceptualization of the work, critical review, and editing of the manuscript. All authors contributed to the article and approved the submitted version.

## Conflict of Interest

The authors declare that the research was conducted in the absence of any commercial or financial relationships that could be construed as a potential conflict of interest. This article is related to a companion perspective: Do We Belittle Essential Tremor by Calling it a Syndrome Rather than a Disease? No. Both Articles were prepared independent of each another.
